# Insights into protein structure using cryogenic light microscopy

**DOI:** 10.1042/BST20221246

**Published:** 2023-11-28

**Authors:** Hisham Mazal, Franz-Ferdinand Wieser, Vahid Sandoghdar

**Affiliations:** 1 Max Planck Institute for the Science of Light, 91058 Erlangen, Germany; 2 Max-Planck-Zentrum für Physik und Medizin, 91058 Erlangen, Germany; 3 Friedrich-Alexander University of Erlangen-Nürnberg, 91058 Erlangen, Germany

**Keywords:** correlative imaging, cryo-EM, cryogenic super-resolution, fluorescence, protein structure and assembly

## Abstract

Fluorescence microscopy has witnessed many clever innovations in the last two decades, leading to new methods such as structured illumination and super-resolution microscopies. The attainable resolution in biological samples is, however, ultimately limited by residual motion within the sample or in the microscope setup. Thus, such experiments are typically performed on chemically fixed samples. Cryogenic light microscopy (Cryo-LM) has been investigated as an alternative, drawing on various preservation techniques developed for cryogenic electron microscopy (Cryo-EM). Moreover, this approach offers a powerful platform for correlative microscopy. Another key advantage of Cryo-LM is the strong reduction in photobleaching at low temperatures, facilitating the collection of orders of magnitude more photons from a single fluorophore. This results in much higher localization precision, leading to Angstrom resolution. In this review, we discuss the general development and progress of Cryo-LM with an emphasis on its application in harnessing structural information on proteins and protein complexes.

## Introduction

**Figure 1.**
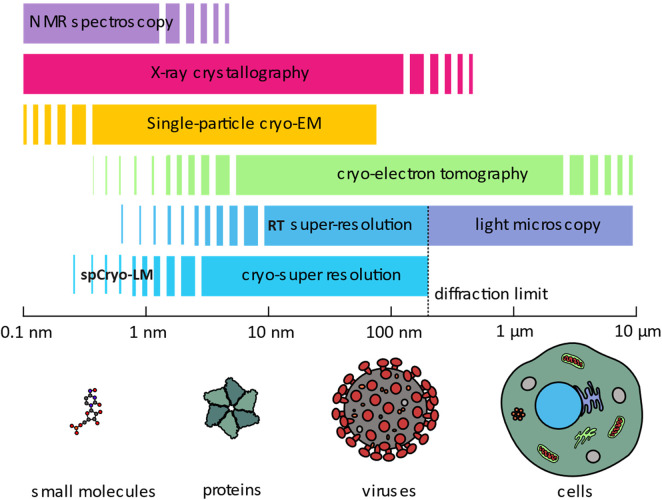
An overview of the resolving capability of different methods for studies in structural biology. Different methods have been developed to investigate biological structures at various scales, ranging from a few Angstroms (small molecules) up to µm scales (cellular structures), not to scale. Light microscopy is unique in its wide coverage of scales, spanning from the Angstrom scale up to the millimeter scale.

‘In the drama of life on a molecular scale, proteins are where the action is’ [1]. Proteins adopt complicated three-dimensional (3D) structures, and assemble into homogenous or heterogenous quaternary structures, ranging from small cellular components up to large assemblies such as viruses. Their 3D arrangement is a part of their dynamic mechanisms of action, governing and orchestrating every aspect of cellular physiology in both health and disease [2–5]. As such, understanding their structure and function has been a major focus in molecular biology. X-ray crystallography and NMR spectroscopy have successfully been used for this purpose, albeit with limitations, especially when dealing with large and complex biological macromolecules [6–12]. More recently, cryogenic electron microscopy (Cryo-EM) has emerged as a revolutionary method, allowing the determination of near-atomic and even atomic resolution structures of isolated macromolecules [13–17] (see Figure 1). The low contrast in this method, however, brings about challenges in identifying individual proteins and target molecules, thus, compromising the quality of attainable structural information in a native cell membrane [18–20], or identifying target molecules *in situ* [21,22].

**Figure 2.**
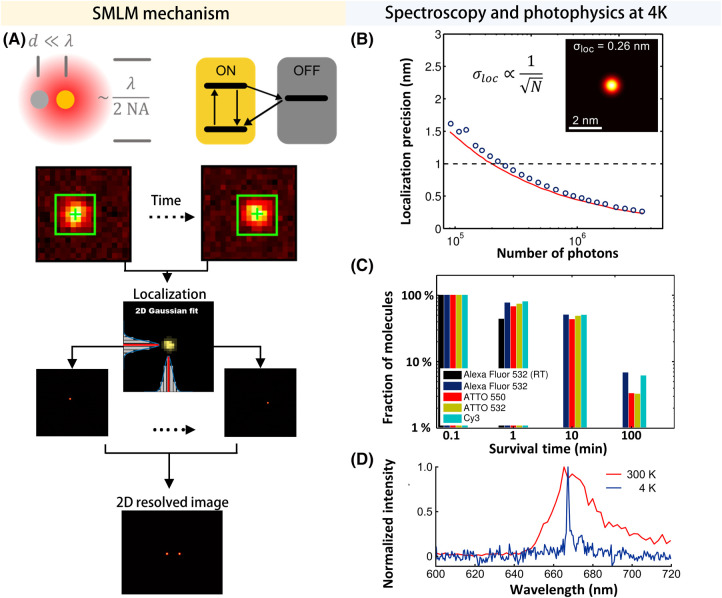
Principle of single-molecule super-resolution microscopy and the advantages of cryogenic operation. (**A**) In SR microscopy based on single-molecule localization, individual fluorescent molecules located within a distance (d) below the diffraction limit of light (λ/2 NA), where λ is the wavelength and NA is the numerical aperture of the objective lens, are imaged one at a time. This process is facilitated by the stochastic transition of the fluorophores from the emissive state (ON) into the dark state (OFF). The PSF of each molecule is then fed into a localization algorithm, such as a 2D Gaussian model, to determine their coordinates with a precision better than the diffraction limit of light. A 2D super-resolved image is typically reconstructed from thousands of localization events [25]. (**B**) Localization precision (σ_loc_) of a single Alexa Fluor 532 molecule at cryogenic temperatures as a function of the number of photons (N). The data represent the standard error of the mean of accumulated localization events. The red line is a theoretical curve based on Ref [32]. The fit to the model shows high localization precision on the order of 2.6 Angstroms (limited by drift correction). (**C**) Cumulative histogram of the survival times for different fluorophores at 4 K compared with Alexa 532 at RT under equivalent illumination conditions. It is evident that photostability is 2–3 orders of magnitude higher at 4 K compared with RT. (**D**) Comparison of a single-molecule emission spectrum (ATTO647N) at RT and 4 K, measured in our laboratory. A close to 10-fold reduction in the linewidth is achieved in the latter case. Panels **B**–**C** adapted from Ref. [69] with permission; copyright 2013 SPIE.

Fluorescence microscopy has been instrumental in studying cellular and sub-cellular structures because it provides molecular specificity. Breakthroughs in single-molecule fluorescence detection [23,24] and manipulation of excitation beams have propelled the super-resolution (SR) era in optical microscopy, enabling investigations at the nanometer scale [25–28,36–41]. A hallmark of single-molecule fluorescence microscopy is the spatial localization of single molecules beyond the diffraction limit of light [29–31]. Here, one ﬁnds the position of each ﬂuorophore by determining the center of its diffraction-limited point-spread function (PSF) with a precision that is dictated by the available signal-to-noise ratio (SNR) (see Figure 2A,B) [32–35]. Hence, a key notion in single-molecule localization microscopy (SMLM) [31] is the ability to turn individual fluorophores on and off. One game-changing approach has been to exploit photo-switchable dyes [25–27,42–47]. Although the attainable resolution in SMLM is theoretically unlimited, in practice it usually does not fair better than 10 nm. First, photobleaching constrains the number of detectable photons, thus limiting the SNR (Figure 2B,C) [32,33,48–53]. Second, Nyquist's sampling theorem and the practical restrictions in labeling density pose a constraint on the achievable resolution [25,54–58]. Nevertheless, there has been a steady push to reach molecular and sub-molecular optical resolution [59–65], bridging the gap between light microscopy and electron microscopy (see Figure 1). These developments are very promising as they promise to shed light on the structure of biomolecules, especially proteins, at high spatial resolution. However, achieving this level of resolution requires a remarkably high degree of mechanical stability against thermal molecular jitter as well as instrumental vibrations and drifts, which has been tamed at room temperature (RT) only through chemical fixation or expansion. To minimize the risk of perturbations in the molecular structure of the sample, scientists have turned to cryogenic measurements, which are known to be compatible with near-native state preservation [66–68]. In this article, we review these efforts with an emphasis on their use in the analysis of proteins and protein complexes.

## Cryogenic light microscopy (Cryo-LM)

Some of the first studies of protein conformational dynamics, in particular the structures underlying their potential energy surface, came from spectroscopic measurements at cryogenic temperatures (CTs) [70–83]. This was done by exploiting the narrowed spectrum of chromophores bound to proteins at low temperatures and following their spectral fluctuations as a function of time. High-resolution spectroscopy of single proteins, for instance, has revealed the dynamics of hydrogen bonds in cofactor binding sites [84]. The advantages of fluorescence studies at CT also gave way to the first single-molecule detection and high-resolution spectroscopy [85,86] as well as the first demonstrations of SR microscopy through spectral selection and localization of individual molecules [30,31,59].

Cryogenic measurements offer two major advantages over RT imaging. First, the superior sample preservation strongly reduces thermal fluctuations and allows spectroscopy and microscopy at very high spectral and spatial resolutions [66,74,84,87–90] (Figure 2D, the data were measured in our laboratory). The second advantage is that photochemistry is considerably slowed down, leading to about three orders of magnitude more emitted photons from a fluorophore than at RT (Figure 2B,C) [69,91–93]. Together with the great asset of specific fluorescence labeling, these features promote the development of cryogenic light microscopy (Cryo-LM) for correlative imaging with other cutting-edge techniques such as Cryo-EM [94–101] that suffer from less specificity. Here, a biological sample is preserved in its near-native state via shock-freezing or high-pressure freezing, where the fast cooling rate preserves water molecules in their random structure, generating amorphous ice (vitreous ice) [67,102–105], which keeps the sample hydrated. This is not the case for the more natural crystalline form of ice, leading to morphological damages to the molecular structure of the sample [67,106]. To maintain vitreous ice, the sample must be kept below the devitrification point which is ∼136 K [104].

**Figure 3.**
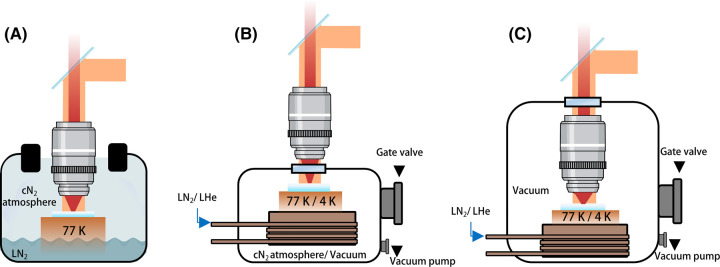
Schematic overview of different cryogenic light microscopes. (**A**) An open-atmosphere LN_2_ optical microscope. These types of setups generally allow easy sample exchange but suffer from condensations as well as severe mechanical and thermal drift. (**B**) A closed high-vacuum/cold N_2_ atmosphere (cN_2_) chamber with a cooling stage. The microscope objective is placed outside the sample chamber. (**C**) A cryostat consisting of a closed high-vacuum optical microscope and a microscope objective placed inside the chamber. This setup showcases exceptional mechanical and thermal stability. Both arrangements **B** and **C**, allows sample exchange in and out of the microscope via a proper cryo-shuttle [102,115].

The simplest way to perform fluorescence microscopy at low temperatures is to cool the sample under ambient pressure [107–109] (Figure 3A). For example, a cold finger can be immersed in a liquid nitrogen (LN_2_) reservoir while maintaining the local surroundings with cold dry nitrogen (cN_2_) atmosphere to prevent ice condensation. This method is easy to use because the sample can be placed under a conventional RT microscope, but the arrangement is prone to condensation and contaminations, both on the sample itself and on the microscope objective. Besides, large temperature variations can give rise to aberrations in the objective. In addition, the setup suffers from severe drifts, which are especially troublesome if one is interested in longer acquisition time [100,110]. Alternatively, enclosed chambers can be used, while the microscope objective sits outside a window to the sample chamber [69,93,99,102] (Figure 3B). Here the sample can be maintained under an N_2_ atmosphere or high vacuum. The latter allows temperatures as low as 4 K by operating with liquid helium (LHe) and offer better performance in terms of sample preservation and mechanical stability [69,93,101,102]. The third arrangement depicted in Figure 3C includes the imaging optics inside the cryostat under vacuum or LHe condition [60,88,111,112]. While all three schemes can use air objectives with numerical apertures (NA) as high as ∼0.9, the last alternative has the advantage of being compatible with using optics with NA > 1 based on solid-immersion lens (SIL) technology [113,114].

For correlative Cryo-LM and Cryo-EM studies, imaging is usually pursued in a sequential fashion. In this process, target-labeled biomolecules are initially mapped using a fluorescent microscope, offering highly sensitive and specific information about their locations. These maps are then employed as references to guide electron microscopy on the same sample, enabling the acquisition of greater informational detail and resolution for a desired region of interest with higher efficiency [94,95,99,110]. For example, Briegel et al. [116] used such an approach to identify the location of chemoreceptor arrays in *C. crescentus* bacterial cell, which were then mapped at higher resolution using cryogenic electron tomography (Cryo-ET). In this genre of applications, one might be satisfied with the diffraction-limited resolution of light microscopy.

**Figure 4.**
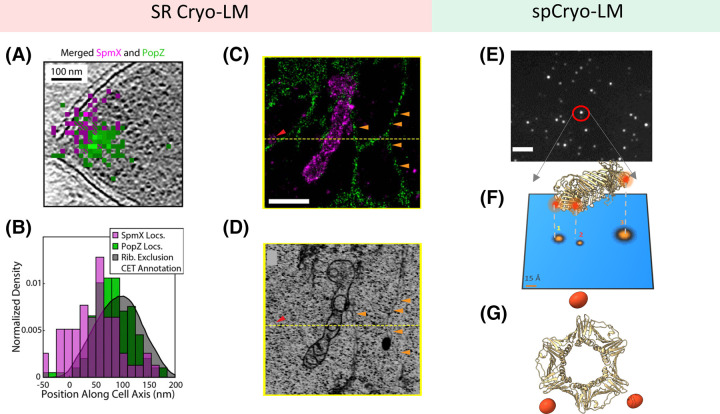
Overview of different super-resolution cryogenic light microscopes and their application to biological samples. (**A** and **B**) Cryo-PALM imaging super-resolves the spatial locations of two model proteins within a frozen-hydrated bacterial cell (PopZ and SpmX, purple and green color, respectively) conjugated to a photo-switchable fluorescent protein (PamKate). This approach combines near-native sample preservation and high photostability of the fluorescent molecules to demonstrate the accurate identification and localization of the two proteins with respect to the bacterial axis with a high spatial precision of 9 nm. This identification was later successfully correlated and validated with the images obtained from Cryo-ET [107]. Panel **A**–**B** adapted from Reference [107] with permission; copyright 2020 PNAS. (**C** and **D**) A near-native whole vitrified cell was imaged at LHe temperature to take advantage of the enhanced photophysics and stability of fluorescent proteins or organic molecules. This approach allowed for the resolution of 3D spatial information and the distribution of protein markers within their ultrastructural context. For instance, by using the highly sensitive localization information of the (ER3-green color), ER protein marker and the outer membrane protein marker of the mitochondria (TOMM20-purple color) (**C**), together with detailed cellular images obtained from FIB-SEM (**D**), a variety of unexpected sphere-shaped ultrastructures were revealed [102]. Orange arrows indicate ER varicosities, and red arrow indicate TOMM20-positive vesicles. The scale bar is 1 µm. Panel **C**–**D** adapted from Reference [102] with permission; copyright 2020 Science. (**E**) Wide-field image of labeled single proteins (scale bar: 5 µm). This method demonstrates spCryo-LM, where the density is chosen to be low enough to only have one single protein or protein complex within the diffraction limit of the optical system. (**F** and **G**) This method able to resolve the configuration of protein complexes with Angstrom-scale resolution (discussed in “Single-particle cryogenic light microscopy” section). The figure depicts a homotrimer of protein PCNA (PDB: 1AXC), where each domain is labeled specifically at the N-termini side with a single fluorophore. The 2D resolve image demonstrate a single projection of the protein in the sample localized with Angstrom scale precision. 2D projections are combined to arrive at the 3D arrangement of the fluorophore on the protein [131].

Cryogenic fluorescence microscopy lends itself particularly well for achieving SR. As a result, several groups have explored this approach on sub-cellular structures (Figure 4A–D). A detailed report on this topic can be found in recent review articles [117,118]. In Table 1, we present an overview of some of the setups used in these efforts with an emphasis on applications of SR microscopy and vitrified samples. In general, the obtained resolution has remained comparable with that achieved at RT, i.e. in the order of 10–150 nm (Figure 1) [102,107–109,117,119,120]. Beside the low NA being used in such microscopes, one can identify three main reasons for the limited resolution: (1) Photophysics at CT, especially photo-switching and photobleaching properties of fluorophores, are not well understood or are insufficient [117,118]. For example, in the case of fluorescent proteins the switching efficiency is reported to be diminished [109,120–124]. (2) The laser power has to be kept low to avoid sample devitrification [120,125,126], leading to low signals. (3) Importantly, most studies have been performed in densely labeled environments, which might hinder one from localizing a single fluorophore with high precision. For example, in Ref. [107] cryogenic photo-activated localization microscopy (Cryo-PALM) was employed using an open atmosphere cryo-stage to determine the spatial location of multiple model proteins with respect to axis of a frozen-hydrated bacterial cell. This yielded ∼10 nm-scale localization precision (Figure 4A,B and Table 1), which was limited by mechanical stability and low laser illumination for fluorescent protein photo-activation. In another recent work [102], the conditions on the laser power and sample stability were improved by mounting the sample on a sapphire disc rather than a carbon film (transmission electron microscope grids). In addition, an enclosed setup operating at LHe temperature provided better mechanical and thermal stability, and it allowed the researchers to exploit the longer dark state of fluorescent protein and fluorescent molecules at high-vacuum and 8 K, reaching a better localization precision (Figure 4C,D and Table 1). A combination of multiple SR methods such as structured illumination microscopy (SIM) and SMLM was employed to super-resolve large cellular structures such as mitochondria and ER in whole vitrified eukaryotic cells with high specificity and sensitivity. Regarding the choice of the coolant medium, LHe is advantageous over LN_2_ as it assures more stable coolant ﬂow and less bubbling although the cost of LHe is much higher. Although LHe provides a significant cryoprotection against radiation damage in electron microscopy [90], its importance for the preservation of biological samples has not been clarified [127,128]. In general, LHe should be able to reduce the thermal jitter of biomolecules more than LN_2_. In our laboratory, we opt for LHe due to its better performance in terms of spectroscopy and photophysics [88,102,129,130]. However, a quantitative investigation and characterization of the photophysics at various temperatures, with and without vacuum has not yet been fully established.

**Table 1:** Cryogenic temperature super-resolution fluorescence microscopy of biological samples

Paper	SR method	Temperature [K]	Condition	Objective	Precision/resolution [x,y]	Precision/resolution [z]	Remarks
[109]	SMLM (Cryo-PALM)	LN_2_	cN_2_	60×, 0.75 NA	170 nm	-	Using Cryostage^2^
[108]	SMLM (Spontaneous blinking)	LN_2_	cN_2_	63×, 0.75 NA	125 nm	-	Using Cryostage^2^
[132]	SMLM (Spontaneous blinking)	LHe	Vacuum	100×, 0.75 NA	∼7 Å	-	1 nm accuracy spCryo-LM (preserved in hydrophilic polymer)
[124]	SMLM (Cryo-PALM)	LN_2_	cN_2_	100×, 0.8 NA	∼ 8 nm (Single molecules)	40 nm (Single molecules)	75 nm in 3D
[133]	SMLM (Cryo-PALM)	LN_2_	cN_2_	100×, 1.3 NA	∼35 nm	-	High NA using cryofluid
[60,131]	SMLM (Spontaneous blinking)	LHe	Vacuum	100×, 0.9–0.95 NA	4–8 Å	-	5–7 Å in 3D, spCryo-LM (preserved in hydrophilic polymer)
[123]	SMLM (Cryo-PALM)	LN_2_	cN_2_	100×, 0.9 NA	9 nm	-	
[134]	SMLM (Cryo-PALM)	LN_2_	cN_2_	100×, 0.8 NA	17 nm		Exceptional stability in an open atmosphere setup
[135]	Spectrum	LHe	Vacuum	Cryo-objective mirrors	1 nm	11 nm	spCryo-LM
[119]	Super-resolution optical fluctuation imaging (SOFI)	LN_2_	cN_2_	50×, 0.9 NA	135 nm		
[120]	SMLM (Cryo-PALM)	LN_2_	cN_2_	100×, 0.75 NA	30 nm		
[136]	SMLM Stochastic optical reconstruction microscopy (STORM)	LN_2_	cN_2_	100×, 0.55 NA with SIL	12 nm		
[107]	SMLM (Cryo-PALM)	LN_2_	cN_2_	100×, 0.9 NA	9 nm	-	Registration error with Cryo-TEM ∼30 nm
[102]	Cryogenic SMLM & SIM	LHe	Vacuum	100×, 0.85 NA	∼2–5 nm	∼25–100 nm	Registration error with Cryo-FIB-SEM ∼40 nm
[137]	Cryogenic 3D-SIM	LN_2_	cN_2_	100×, 0.9 NA	210 nm	640 nm	Obtained at 488 nm laser excitation
[138]	Cryogenic confocal microscope	LN_2_	cN_2_	100×, 0.75 NA	290 nm	1150 nm	ZEISS LSM 900 confocal microscope equipped with an Airyscan 2 detector
[139]	Cryogenic super-resolution radial fluctuations (Cryo-SRRF)	LN_2_	cN_2_	0.9 NA	∼100–200 nm	-	EM Cryo CLEM (Leica Microsystems)

## Single-particle cryogenic light microscopy (spCryo-LM)

A fundamental challenge in SR microscopy is achieving a very high-density labeling to satisfy the Nyquist–Shannon sampling theorem [57]. For example, to be able to image all parts of a dense two-dimensional (2D) structure at a resolution of a few tens of nanometers, several thousands of fluorophores must be localized within a diffraction-limited spot. For a sample that is extended in the third dimension, the number scales accordingly, making it a daunting task to resolve cellular structures with a true resolution in the order of a few tens of nm. Even if this were to be realizable, one would then require a sufficiently performant photo-activation to ensure that only one fluorophore is on at any given time in order to achieve high localization precision and structural resolution. Nevertheless, SR Cryo-LM can be exploited to resolve the 3D configuration of isolated finite-sized nanostructures at Angstrom optical resolution. As depicted in Figure 4E–G, here one chooses a sparse coverage of the nanostructures to avoid having more than one per PSF. Moreover, each subdomain of interest is conjugated with a single fluorophore. If the number of subdomains is not too large, one can localize each fluorophore individually at Angstrom resolution, thus, deciphering the stoichiometry and assembly of the nanostructure at hand. We shall refer to this technique as single-particle cryogenic light microscopy (spCryo-LM), which we choose as the main focus of this review. Considering the recent emergence of this approach, the article remains somewhat biased on the work from own laboratory.

### Photoblinking at 4 K

SMLM relies on mechanisms that allow one to image one molecule at a time. The pioneering works in RT SR microscopy used photo-activation of synthetic dyes [43] or fluorescent proteins [26,44,45]. In principle, the same techniques can also be used in spCryo-LM [60,131], but as mentioned earlier, knowledge of these phenomena at CT is still limited. In our laboratory, we have chosen to use naturally occurring stochastic photoblinking of organic dyes [25]. Fluorescence intermittency in these molecules has been extensively characterized at RT and has often been found to follow non-exponential probabilities. This behavior has been attributed to transitions to trapped states, which generation can depend on the environment of the surrounding material and temperature as well as the excitation wavelength and intensity. Despite several vigorous studies, many questions remain open in this field [143–150].

Investigations of photoblinking of organic molecules at low temperatures are scarce [151]. At LHe temperature, however, we find several blinking behaviors, such as exponential and power law for several conventional dyes (ATTO, Cy, Alexa) [130,152,153]. As a physical rule of thumb, one can argue that under ambient conditions transitions from the triplet state back to the ground state are typically mediated by collisions with singlet oxygen [151,154,155]. The abundance of oxygen ensures a fast fluorescence recovery and short off-times in this case. At low temperatures and in high vacuum, diffusion of singlet oxygen is reduced, leading to longer off-times, which is favorable for single-molecule localization microscopy.

In general, to resolve N fluorophores unambiguously within a diffraction-limited spot, we require an on–off ratio smaller than 1/N. In addition, the frame rate of the camera needs to be faster than the average off-time to minimize the probability of overlapping contributions from many molecules in a single image. It is not straightforward to predict the on- and off-times of fluorophores at CTs from their values in solution, and common strategies cannot be directly used to engineer them. Typically, the off-on ratio ranges between 5 to 30, and strongly depend on the nano-enviroment, as well as illumination power [131,152]. Different setups have reported spontaneous blinking at 4 K, but experiments operating at LN_2_ and open atmosphere have not been successful in achieving high blinking ratios. As a result, they have been limited in the number of collection photons and the attainable localization precision [107,118]. Modulating the blinking behavior at CT is still not explored.

### Identification by brightness

**Figure 5.**
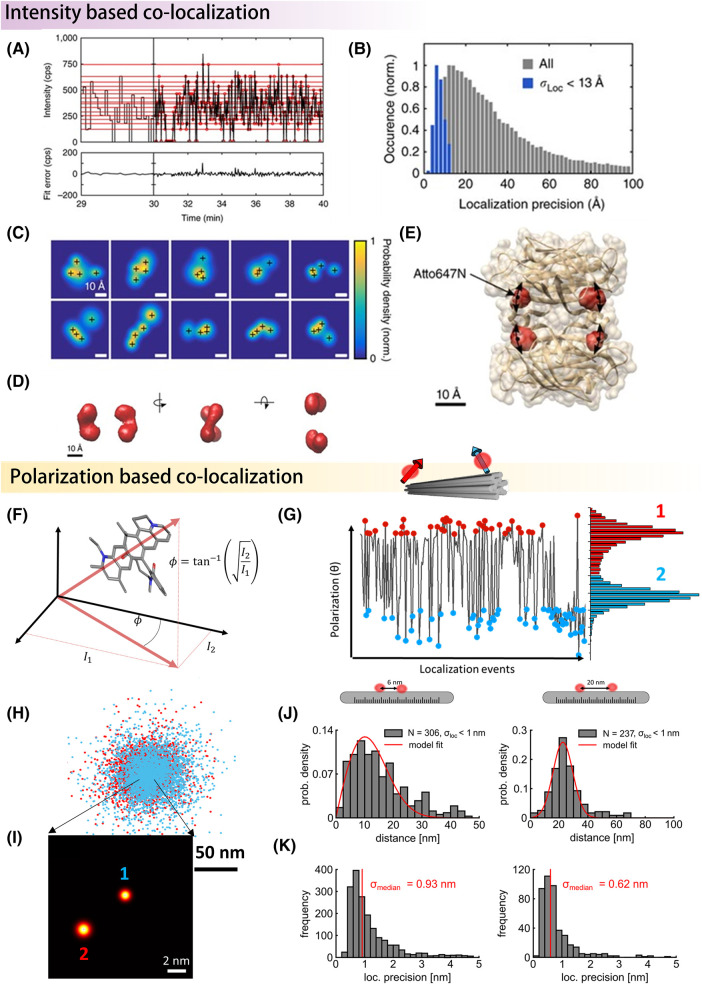
single-particle cryogenic super-resolution approaches. (**A**) Intensity-based co-localization approach. Here, intensity levels in a blinking time trace are used to annotate and localize each fluorophore separately. The example depicts a time trace of four fluorophores on a streptavidin protein conjugated to four labeled biotin molecules. The time trace exhibits 2^4^ distinct levels, which correspond to various combinations of the four fluorophores due to their distinct local environments [60]. After fitting the data with a proper model, only the lowest four levels are utilized for annotating and localizing the fluorophores over time. (**B**) The overall localization precision is obtained from thousands of particles (gray color). The blue area highlights the molecules with high localization precision. (**C**) 2D resolved images obtained from localizing each identified fluorophore based on the intensity trace, showing different particles at different projections. By exploiting single-particle analysis algorithms borrowed from Cryo-EM, distinct 2D resolved projections (**C**) are reconstructed to reveal the 3D spatial configuration of the fluorophores (**D** and **E**, PDB: 1STP) [60]. (**F** and **G**) The second and more robust approach to fluorophore annotation involves exploiting their fixed dipole orientation at CT (**F**). The polarization time trace in (**G**) is obtained by measuring a DNA origami structure conjugated with two fluorophores at a fixed distance (see inset), and depict a short part of the on events cropped from a long trajectory. One finds two distinct populations of polarization, which reflect the appearance of the two fluorophores as they blink over time. By collecting all the raw localization events for each fluorophore separately, a 2D super-resolved image can be reconstructed (as shown in **I**, depicted in **H**). Employing this approach enables resolving variety of distances with sub-nanometer localization precision (**J**) [152]. The Inset depict the designed distance on a DNA origami substrate. The top histograms depict the distance distribution, and bottom histograms depict the localization precision, respectively (**K**). Panels **A**–**E** adapted from Ref. [60] with permission; copyright 2017 Nature Springer. Panels **G**–**K** adapted from Reference [152] with permission; copyright 2020 ACS photonics.

The early work on spCryo-LM demonstrated co-localization of two organic fluorophores on a DNA backbone, reaching sub-nanometer accuracy [132]. The method was then extended to resolving two fluorophores bound to the C termini of the cytosolic GtCitA PAS protein domain and four fluorophores bound to the four biotin sites of single streptavidin molecules [60]. This first application of spCryo-LM reached the remarkable 3D resolution of 5 Angstrom for sites distanced by ∼2 nm (Figure 5A–E). This method was named cryogenic optical localization in 3D (COLD) [60]. Here, proteins were embedded in a hydrophilic polymer at LHe temperature and by exploiting the slow stochastic blinking of organic fluorophores, each was identified based on its intensity level (Figure 5A). In the most general case, the time traces are expected to show only *N* discrete jumps corresponding to the step-wise photoblinking of *N* identical ﬂuorophore per particle. However, variations in orientation, local environment and quantum efﬁciency leads to 2^N^ combinations of the on/off-state signal levels. Individual molecules are addressed by sorting imaging frames that correspond to each of the *N* lowest levels and taking their average for localizing each level separately.

Upon the successful localization of the fluorophore positions conjugated to a target molecule, it is straightforward to generate the 2D resolved images, whereby a 2D Gaussian function is assigned to each localized fluorophore with a width given by the respective localization precision. Due to the 3D random orientations of individual proteins in the sample, a given structure gives rise to a wealth of 2D projections (Figure 5C). Assuming that all structures are intrinsically identical, one can follow a protocol similar to that used in single-particle reconstruction in electron microscopy [14,156]. Here the 2D resolved images are fed to a maximum-likelihood algorithm, which reconstructs the 3D structure in an iterative manner (Figure 5D,E). The reconstructed model can generally be improved by filtering the 2D data sets, for example, by filtering based on the localization precision (Figure 5B), number of photons, maximum/minimum resolved distance, number of polarization states, etc.

**Figure 6.**
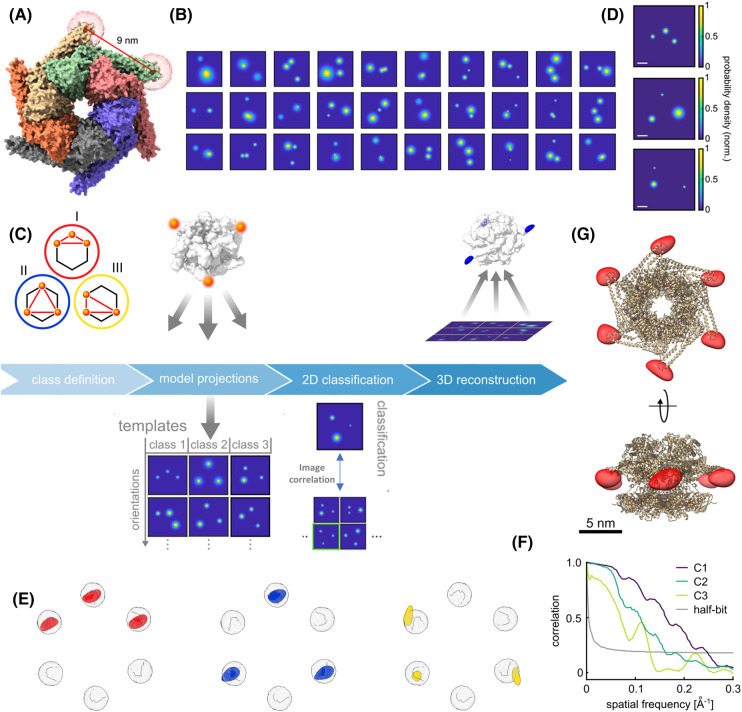
Single-particle classification and reconstruction in a model-system. (**A**) Crystal structure of hexamer protein, ClpB (PDB: 1QVR). The labeling positions and the distance between them are marked. (**B**) 50% labeling efficiency of the protein inherently yields three different classes of different configurations and distances due to the C6 symmetry of the hexamer (**C**). (**B**) Examples of 2D super-resolved particle images in a 30 nm × 30 nm box with 0.15 nm pixel size emerge from the heterogeneous particles in the sample. (**C**) A strategic pipeline for classifying the images obtained in (**B**) to three classes. An approach based on template matching is employed here. Initially, an extensive library of images is generated for each class, encompassing all possible 3D orientations. Subsequently, each experimental image is cross-correlated with the image library of all classes. The experimental images are assigned to the class that yielded the highest correlation score. (**D**) A near-top view projection of each class. The images for each class are subsequently employed for separate 3D reconstruction. (**E**) The reconstructed fluorophores’ positions (red, blue, yellow) align with their expected theoretical accessible volumes (gray spheres). (**F**) Fourier shell correlation curves of the reconstructed ﬂuorophore volumes. The intersection with the half-bit criterion determines resolutions of 4.0, 7.9 and 6.4 Å, respectively. (**G**) By combining the partially resolved classes, a full structure can be resolved. Panels **D**, **E**, **F** and **G** adapted from Reference [131] with permission; copyright 2022 eLife.

Upon successful co-localization and assignment of fluorophore positions within a particle, a quantitative value that estimates the quality of the model reconstruction can be obtained using the Fourier shell correlation (FSC) in 2D or in 3D [157,158]. FSC simply calculates the correlation between the two half data set reconstructions. The resolution is then determined by ﬁnding the point of intersection of the FSC curve with the curve of a resolution criterion, such as the half-bit criterion (Figure 6F) [159].

### Identification by polarization

The intensity-based co-localization approach discussed above suffers from low yield because in most cases, the blinking traces are not sufficiently well resolved. A more robust and efficient scheme exploits polarization differences from individual fluorophores on a given particle, see Figure 5F–K [152], taking advantage of the fact that contrary to RT experiments, fluorophores do not rotate and possess fixed dipole orientations. Unlike fluorescence brightness, which is subject to temporal fluctuations due to transitions between nearby levels (Figure 5A), polarization angles can be separated more clearly, thus reducing errors in measurements (Figure 5G).

A simple method to measure the molecular orientation is to split the emitted light with a single polarizing beam splitter to two channels with orthogonal polarizations. The polarization in the image plane is then determined from the ratio of the intensities registered in the two channels, which yield an angular interval θ ɛ [0°, 90°] (Figure 5F,G). This approach, which was termed polarCOLD, was first demonstrated on DNA origami structures ranging from 6 nm up to 95 nm with high localization precision (Figure 5J,K) [152,153]. In Figure 4F, we present an example of a homotrimer protein complex PCNA labeled with three ATTO647N dyes at N-termini sites [131]. The three distinct populations of polarization trajectories were used to locate the three binding sites. A 2D resolved image was generated from each trajectory by clustering the localization of polarization populations separately. The resulting images were then used to solve the 3D information.

Despite the extended range in the polarization space, resolving larger numbers of labels per particle becomes increasingly challenging (low yield) because of the higher probabilities of several overlapping polarizations. Currently, we estimate that about six emitters per particle can be addressed comfortably based on the angular width of each polarization distribution [131]. Application of more sophisticated analysis methods, e.g. based on machine learning or imaging in 3D, promises to push this limit further. As another way to address this limitation, we reduced the labeling efficiency of the fluorophores such that the majority of the complexes contained 2–4 molecules per complex only. As an example, a protein disaggregation machine composed of six identical subunits, ClpB, was labeled at 50% labeling efficiency by attaching an ATTO647N dye specifically on its M domain. Given the hexagonal symmetry of the structure, three distinct classes of configurations are expected (Figure 6A–C). To sort these various classes and to demonstrate the ability of this approach for studying heterogenous samples, a pipeline based on supervised template matching was implemented to classify each 2D projection (Figure 6C). Figure 6D–F displays the remarkable success of the method in identifying three different configurations within the same sample with high confidence. After solving their 3D structures separately, they were merged together to obtain the full 3D configuration of the six labeling sites on the ClpB protein and to decipher the symmetry of the protein complex at high resolution (Figure 6F,G) [131]. In the case of an unknown sample, this approach might require some prior knowledge on the number of monomers per molecule, which can be obtained from several biochemical assays such as native gel, gel chromatography or even simple negative stain electron microscopy.

Quantitative classification approaches have also been demonstrated in the field of single-particle SR microscopy at RT mainly assuming symmetric models and applied to large protein complexes such as the nuclear pore complex [160–165]. For example, Curd et al. [162] developed a pipeline that finds the symmetry of the protein complex based on a pairwise distance histogram. Heydarian et al. [166] used an all to all registration scheme to combine a classified projection into one average data set, which resolved the complete structure. However, this approach works mainly on a close to top view images, in case of 2D imaging approach. A 3D version of the same scheme was developed later which considers the information from the 3 coordinates x, y and z. This approach allows resolving particles with different orientations but it assumes a homogenous subset of the molecules, i.e. a single conformation. For example, this approach was recently applied to resolve several conformation of the PIEZO1 protein in a chemically fixed cell membrane [167]. We remark that the complexity of the orientation problem can be reduced significantly by confining the protein to a single orientation, e.g. via tethering to a surface such as DNA hybridization or similar approaches [168].

## Future directions

Structural biology faces new challenges as it ventures into the native cellular environment targeting smaller proteins and their complexes. It is to be expected that all existing imaging spectroscopy methods continue to improve both on the hardware and the analysis sides, especially taking advantage of powerful machine learning algorithms. The Angstrom localization precision obtained in Cryo-LM already surpasses the fundamental resolution limit of fluorescence microscopy, which is posed by the physical size of the fluorophore rather than the laws of physics. It is, thus, interesting to develop fluorescent dyes with minimal extension. Moreover, design and synthesis of fluorophores with optimized switching capabilities at low temperature would strongly benefit Cryo-LM.

It is to be born in mind that the community of SR microscopy considers the individual fluorophores to be independent. While this is a reasonable assumption at distances larger than 10 nm and at RT [55], molecules can undergo coherent and incoherent dipole-dipole coupling at very small distances such that the locations of emission and absorption no longer coincide. This would smear the localization precision at the nanometer scale. The best known case of incoherent coupling is that of fluorescence resonant energy transfer (FRET), where energy is transferred from a donor molecule to an acceptor molecule at distances less than ∼10 nm [169]. It is important to keep in mind that the less known homoFRET can also take place between molecules of the same species. Moreover, as dephasing is reduced at CTs, coherent coupling between molecules can lead to the hybridization of the energy levels and again delocalize emission and absorption over the extent of the molecular ensemble [59]. So far, we have not confronted restrictions from such dipole-dipole couplings, but future studies are needed to quantify their role. Indeed, combination of Cryo-LM and cryogenic laser spectroscopy would provide more information about the nature of the coupling, which depends on the fluorophore orientation and distance much in analogy with the analysis of NMR spectra.

The currently achieved SNR and Angstrom precision of spCryo-LM is already capable of providing pivotal solutions for quantitative structural analysis of small proteins as well as large protein complexes and aggregates. In particular, crucial information about protein assembly such as configuration and symmetry as well as conformational changes can be obtained by labeling various domains. Moreover, protein–ligand interactions can be investigated by labeling ligand molecules such as biotin or ATP. Novel algorithms based on deep learning [170,171] also promise to increase the number of fluorescent molecules that can be identified per particle and will enhance the measurement yield by improving particle classification.

A specially promising line of study concerns the conformation and clustering states of membrane proteins in their native environment. Indeed, an estimated 20% of the human genome encodes membrane proteins and many of them are potential drug targets [172]. In current experiments in our laboratory, we vitrify and preserve biological samples via rapid freezing [67,173], allowing us to perform spCryo-LM on membrane proteins in their natural environment. As discussed previously, this approach has been successfully implemented in different types of microscopes [102,107,119,120] (see also Table 1). In this case, membranes can be prepared by cell unroofing or generation of cell-derived membrane vesicles [174,175]. Such investigations would ideally complement many existing techniques such X-ray crystallography, NMR, and Cryo-EM, which do not perform well on membrane proteins in their native environment, e.g. as a result of high background signal from the lipid environment [18]. We can also expect spCryo-LM to assist in solving many dynamic biomolecular structures and dissect the full energy landscape of protein machines.

A naturally emerging exciting avenue of spCryo-LM is a combination with single-particle Cryo-EM. Although these techniques are referred to as ‘single-particle’ methods, the high-resolution structural information only becomes available after averaging over many particles. In Cryo-EM, several hundreds of individual 2D particle images are averaged to increase the SNR for the classification procedure [176–178] because each image delivers only a small amount of contrast. After aligning, averaging and classification in 2D, in most cases 3D atomic level resolution is achieved from nearly 10–20% of the identified particles as a result of particle heterogeneity or particle damage at the air-water interface [179,180]. As a result, the method still faces challenges in identifying structural heterogeneities caused by mobile domains and intrinsic distributions in assemblies [181–188]. The higher SNR in spCryo-LM, on the other hand, reduces the number of necessary averages by about two orders of magnitude because each 2D projection directly contributes to the 3D reconstruction process. Thus, data from spCryo-LM could massively enhance the yield, reduce error and increase resolution in single-particle Cryo-EM by providing ground truth annotation. In addition to single-particle analysis, high localization precision of fluorescent tags will aid in determining the spatial location of proteins *in situ*, in combination with the rapidly growing field of Cryo-ET [21,102,107,124,189–191]. For this task, however, one has to achieve high labeling densities and tame the background fluorescence, which demands a deeper understanding of the photophysics at CT.

## Perspectives

- Cryogenic light microscopy is an emerging high-end technology, which holds great promise in shedding light on the structure of biomolecular entities such as proteins, protein complexes and membrane proteins in their native state within the context of cellular ultrastructure.
- Currently, the method can achieve Angstrom-level resolution for soluble proteins and protein complexes. Work is under way to extend this method to membrane proteins, where other structural biology techniques encounter challenges.
- With a better understanding of the photophysics at CT, it will become possible to conduct *in situ* correlative studies using both light and electron microscope techniques at Angstrom-scale resolution. This will enable the dissection of cellular components and protein structures and will provide unprecedented insight into their physiological roles.

## Open Access

Open access for this article was enabled by the participation of Max Planck Digital Library in an all-inclusive *Read & Publish* agreement with Portland Press and the Biochemical Society under a transformative agreement with MPDL.

## Abbreviations

cN_2_: cold dry nitrogen
CTs: cryogenic temperatures
Cryo-EM: cryogenic electron microscopy
Cryo-ET: cryogenic electron tomography
Cryo-LM: cryogenic light microscopy
Cryo-PALM: cryogenic photo-activated localization microscopy
FSC: Fourier shell correlation
LHe: liquid helium
LN_2_: liquid nitrogen
NA: numerical apertures
PSF: point-spread function
RT: room temperature
SIL: solid-immersion lens
SIM: structured illumination microscopy
SMLM: single-molecule localization microscopy
SNR: signal-to-noise ratio
SR: super-resolution
spCryo-LM: single-particle cryogenic light microscopy
